# Radar Signal Processing and Its Impact on Deep Learning-Driven Human Activity Recognition

**DOI:** 10.3390/s25030724

**Published:** 2025-01-25

**Authors:** Fahad Ayaz, Basim Alhumaily, Sajjad Hussain, Muhammad Ali Imran, Kamran Arshad, Khaled Assaleh, Ahmed Zoha

**Affiliations:** 1 James Watt School of Engineering, University of Glasgow, Glasgow G12 8QQ, UK; f.ayaz.1@research.gla.ac.uk (F.A.); b.alhumaily.1@research.gla.ac.uk (B.A.); sajjad.hussain@glasgow.ac.uk (S.H.); muhammad.imran@glasgow.ac.uk (M.A.I.); 2 Department of Electrical and Computer Engineering, College of Engineering and Information Technology, Ajman University, Ajman P.O. Box 346, United Arab Emirates; k.arshad@ajman.ac.ae (K.A.); k.assaleh@ajman.ac.ae (K.A.); 3 Artificial Intelligence Research Centre, Ajman University, Ajman P.O. Box 346, United Arab Emirates

**Keywords:** human activity classification, radar domain representations, deep learning, computational cost, transfer learning

## Abstract

Human activity recognition (HAR) using radar technology is becoming increasingly valuable for applications in areas such as smart security systems, healthcare monitoring, and interactive computing. This study investigates the integration of convolutional neural networks (CNNs) with conventional radar signal processing methods to improve the accuracy and efficiency of HAR. Three distinct, two-dimensional radar processing techniques, specifically range-fast Fourier transform (FFT)-based time-range maps, time-Doppler-based short-time Fourier transform (STFT) maps, and smoothed pseudo-Wigner–Ville distribution (SPWVD) maps, are evaluated in combination with four state-of-the-art CNN architectures: VGG-16, VGG-19, ResNet-50, and MobileNetV2. This study positions radar-generated maps as a form of visual data, bridging radar signal processing and image representation domains while ensuring privacy in sensitive applications. In total, twelve CNN and preprocessing configurations are analyzed, focusing on the trade-offs between preprocessing complexity and recognition accuracy, all of which are essential for real-time applications. Among these results, MobileNetV2, combined with STFT preprocessing, showed an ideal balance, achieving high computational efficiency and an accuracy rate of 96.30%, with a spectrogram generation time of 220 ms and an inference time of 2.57 ms per sample. The comprehensive evaluation underscores the importance of interpretable visual features for resource-constrained environments, expanding the applicability of radar-based HAR systems to domains such as augmented reality, autonomous systems, and edge computing.

## 1. Introduction

Human activity recognition (HAR) is now fundamental for applications in medical rehabilitation [1,2], intelligence security [3], and ambient-assisted living [4,5]. Traditionally, HAR has relied on data from video surveillance [6,7], infrared cameras [8,9], wearable sensors [10,11], and ambient sensors [12]. Ambient sensors such as motion detectors, energy consumption monitors, and temperature sensors have been widely used in smart home environments to infer human activities. For instance, they can detect door openings, monitor energy usage, and track environmental changes to provide insights into human behavior.

However, each of these methods has limitations: video surveillance experiences poor accuracy in low-light conditions due to environmental factors and monitoring distance. At the same time, infrared cameras are sensitive to temperature changes and struggle to detect radial motions. Meanwhile, wearable sensors have not seen widespread adoption due to their intrusiveness and limited battery life. Similarly, ambient sensors often rely on extensive infrastructure for deployment and operation, which can limit their scalability and adaptability to dynamic environments. Additionally, they may face challenges in terms of reliability and precision because their indirect measurements can be influenced by environmental factors or ambiguous activity patterns, making it difficult to accurately interpret complex human behaviors.

In contrast, radar sensors [13,14,15,16,17], which have been increasingly used in recent decades, offer solutions to these challenges, particularly in short-range indoor applications. Radar sensors effectively localize and track human movements, recognize various behaviors, and even monitor vital signs [18,19,20] without the need for individuals to carry the devices. With their high penetration and privacy protection capabilities, radar sensors offer a versatile and non-intrusive option for HAR in sensitive environments, such as bedrooms and bathrooms, addressing the functional and privacy limitations of other types of sensors.

Radar echoes typically contain information regarding time, range, and Doppler frequency. However, researchers [16,21,22] often perform time-frequency analysis on radar echoes to obtain spectrograms with micro-Doppler (μ-D) features, which are then used in HAR for recognition and classification. Radar spectrograms offer a unique visual representation of human activity, positioning them as an alternative to conventional image data. Although other radar data representations, such as the Time-Range (TR) domain based on range-fast Fourier transform (FFT) [23,24], are available, radar-based representations remain a popular choice for HAR because of their ability to capture the distinct movement characteristics of individual body parts.

In this study, we explored multiple representations of the radar domain to maximize information extraction from radar echoes. The first representation is a two-dimensional (2D) TR domain map generated using the range-FFT. In addition, we used two frequency–time-based approaches, namely the short-time Fourier transform (STFT) [25] and the smoothed pseudo Wigner–Ville distribution (SPWVD) [26]. STFT provides apparent and interpretable features in the frequency–time domain, although it is limited by a fixed-length window function, creating a trade-off between time and frequency resolution. In contrast, SPWVD offers high frequency–time resolution and effectively reduces cross-term interference, allowing for the precise representation of μ-D features. Although SPWVD resolution exceeds that of STFT, it requires significantly more processing time, making it less suitable for real-time applications, which is a key consideration in this study.

The effectiveness of HAR is influenced by both the creation of diverse radar representations and the choice of feature extraction techniques. Generally, feature extraction methods fall into two categories: manual and automated through deep learning models [13]. However, manual extractions are prone to interference from noise, require specialized knowledge, and fail to isolate high-level discriminative details from radar-based representations, leading to less efficient results. In response, deep learning-based HAR approaches have emerged, which use convolutional neural network (CNN) models to enhance feature extraction by capturing a broader set of features from radar signals. CNNs have significantly improved the ability to autonomously learn and distinguish complex data patterns. CNNs are particularly effective in processing image data, including radar-generated maps, because they can perform both feature extraction and classification. This dual functionality has revolutionized fields such as image recognition and computer vision [13].

A groundbreaking study introduced the first innovative CNN model for document recognition [27]. However, it was pioneering work that brought CNN algorithms into the spotlight. Another impressive work by [28] is their CNN architecture, which achieved a top-one error rate of 37.5% in the ImageNet challenge in 2012. Due to this significant breakthrough, several CNN architectures have been developed, including VGGNet, MobileNet, and ResNet. Due to their excellent performance in image classification, these architectures have also been applied to the processing of radar domain representations for HAR classification. These 2D radar representations, such as TR maps and TD maps with μ-D features, act as data inputs for HAR in a manner similar to the way images from vision sensors are used. Although radar and image data are different, these radar spectra can be analyzed in a manner similar to that of visual data, enabling activity recognition based on identifiable patterns. However, training a CNN from scratch requires a large amount of data, which is often rare in specialized applications such as radar-based HAR, resulting in overfitting or underfitting.

To overcome this challenge, we employ a transfer learning (TL) approach [29,30,31]. TL allows the use of pre-trained models on large datasets, thus enabling the adaptation of existing CNNs to new tasks. This strategy significantly reduced the need for large amounts of data and accelerated the training process. Therefore, our study leveraged well-known CNN architectures, such as VGG-16 [32], VGG-19 [33], ResNet-50 [32], and MobileNetV2 [34], which were chosen for their demonstrated effectiveness in image-based learning tasks, which are well suited for processing radar-generated images. We fine-tuned our training samples on these four pre-trained architectures to optimize the HAR system for better recognition accuracy and fast real-time prediction, which is essential for critical applications such as fall detection.

The VGG model, which is known for its deep architecture, provides powerful feature extraction capabilities. ResNet-50 introduced residual learning to solve the gradient vanishing problem, thereby facilitating the training of deeper networks. MobileNetV2 uses depth-wise separable convolutions, which improve computational efficiency by processing each input channel separately and then combining the feature maps with 1 × 1 convolutions. Additionally, MobileNetV2 integrates reverse residual connections and a modified residual link to learn more complex features while maintaining efficiency.

In this study, we suggest analyzing the performance of various preprocessing techniques and CNN architectures, specifically focusing on their potential application in edge-computing scenarios with limited computational resources. By optimizing these techniques with real-time processing, this approach aims to enhance the accessibility and effectiveness of HAR systems in real-world environments. Our proposed framework explores three different preprocessing techniques and four CNN models, resulting in 12 unique data preprocessing and model combinations. We aim to evaluate their recognition accuracy and efficiency, ultimately identifying the most promising combination for potential deployment on resource-constraint devices. Our contributions can be summarized as follows:

- **Evaluation of radar 2D domain techniques:** we empirically evaluated range-FFT-based time-range (TR) maps and time-Doppler (TD) maps generated using STFT and SPWVD, quantifying their computational efficiency in real-time HAR systems.
- **Optimizing models with transfer learning (TL):** we evaluated the performance of state-of-the-art CNN architectures, including VGG-16, VGG-19, ResNet-50, and MobileNetV2, to improve the accuracy of the proposed HAR system using TL methods.
- **Performance and computational analysis of model-domain pairs:** We conducted a comprehensive analysis of 12 model-domain pairs, focusing on real-time performance to optimize the balance between accuracy and computational efficiency (preprocessing, training, and inference times). The analysis is also extended to performance metrics beyond accuracy, such as recall, precision, and F1 score, which are critical to evaluating effectiveness in real-world applications.

**Figure 1:**
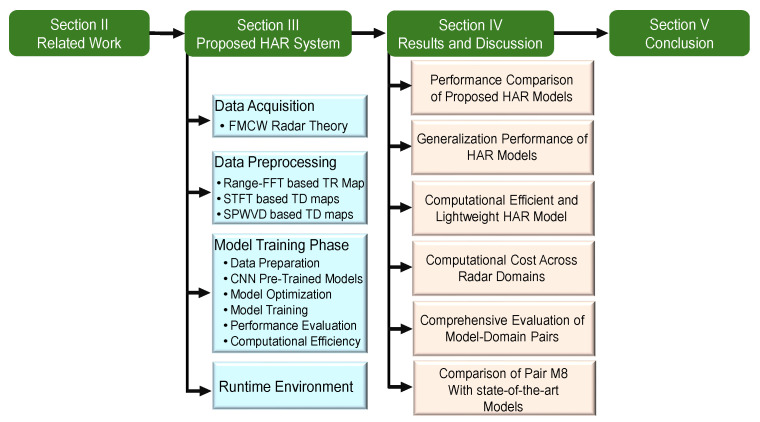
Detailed flow diagram illustrating the structure of the paper sections and content.

The remainder of this paper is structured as follows: In Section 2, we outline the related work. Section 3 presents an in-depth description of the radar-based HAR approach, covering the radar technology, the dataset, the preprocessing techniques, and the CNN architecture used. Section 4 presents a comparative evaluation of different combinations of radar data preprocessing and CNN models. Finally, Section 5 summarizes the main findings and contributions of this study and suggests possibilities for future research. The methodological flow of this study is illustrated in Figure 1.

## 2. Related Work

Researchers have increasingly turned to radar for non-intrusive activity monitoring. Over time, many approaches and techniques have been developed to improve the accuracy of such monitoring, particularly given the scarcity of comprehensive radar datasets. Different radar domains derived from radar echoes have become pivotal in training classification models. Although conventional machine learning methods have been explored in previous studies [35,36,37], recent advances have shifted towards deep learning applications on radar datasets [13,38]. To address the challenge of limited data, we applied transfer learning methods [39,40], leveraging the capabilities of models pre-trained on large datasets.

In contrast to prior research that primarily focused on single radar domains or models, this study explored three distinct radar domain representations derived from the fast Fourier transform (FFT): time-range (TR), short-time Fourier transform (STFT), and smoothed pseudo-Wigner–Ville distribution (SPWVD) maps. These domains were analyzed in conjunction with various state-of-the-art neural network configurations. As shown in Table 1, existing studies often rely on only one or two radar domains, such as TR or STFT-based time-Doppler (TD), for activity recognition. Studies such as [23,24,25,26,38] partially addressed this by incorporating multiple domains; however, they failed to integrate all three domains (TR, STFT, and SPWVD) for comprehensive radar representations. Similarly, studies [16,41,42,43] remain limited to TD domains. Another critical gap in these studies, as illustrated in Table 1, is the lack of attention to the data preprocessing time and overall computational efficiency. Although radar-based HAR is often considered for real-time applications, none of the existing studies have reported the time required for preprocessing the raw radar data. Although these approaches effectively capture specific features, they fail to exploit the complementary strengths of multiple radar domains and overlook the practical constraints of real-time deployment such as computational demands.

Deep learning (DL) models, particularly convolutional neural networks (CNNs) and long short-term memory (LSTM) networks, are widely used for radar-based HAR for activity classification. Studies [16,23,24] relied mainly on CNNs with TR and STFT domains as inputs, whereas researchers from [26,38] combined CNNs with LSTMs to capture spatiotemporal features using STFT and SPWVD domains as input, respectively. Although these models have shown strong performance, they often require large datasets and significant computational resources, which may not be practical for radar-based HAR applications. In this study, we employed the same model architectures as those illustrated in [24,43] for direct comparison. Using the STFT-based TD domain, we achieved recognition accuracies of 84.90% and 95.16%, respectively. However, these models resulted in higher computational time, particularly during the training and prediction of the model. This underscores the trade-off between the accuracy and computational efficiency in these approaches, which is critical for resource-constrained applications.

Transfer learning (TL) approaches, which are particularly well suited for handling small radar-based HAR datasets and reducing computational demands, remain underexplored in the studies summarized in Table 1. For instance, only a few studies, such as [25,42], have employed TL methods that rely on a single pre-trained model. In contrast, our study integrated four different pre-trained models, namely VGG-16, VGG-19, ResNet-50, and MobileNetV2, alongside CNN and LSTM architectures to provide a comprehensive comparison of DL approaches. This inclusion highlights the versatility and efficiency of TL for addressing the challenges posed by small datasets and computational constraints. By achieving a higher recognition accuracy and efficient time measurements across multiple radar domains, these models demonstrate their suitability for resource-constrained scenarios, particularly in real-time radar-based HAR applications.

By systematically analyzing 12 model–domain pairs (MDPs), we aimed to provide deeper insight into their effectiveness and contribute to the advancement of radar-based HAR systems. Our study improves accuracy while evaluating four distinct performance metrics along with computational costs. To the best of our knowledge, this comprehensive analysis of MDPs in a single study, focusing on both computational costs and overall classification accuracy, is unmatched in the field. Consequently, our work is highly relevant to the ongoing development of radar-based HAR technologies, and it sets a valuable benchmark for future research and development.

## 3. Radar-Based HAR System

**Figure 2:**
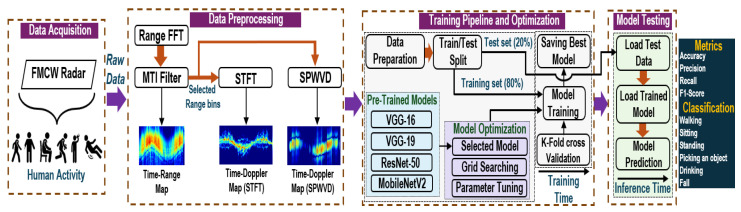
Radar-based HAR system depicting the workflow from data acquisition to radar maps’ generation, along with state-of-the-art neural networks.

The system model for this study is shown in Figure 2, which outlines a comprehensive process that begins with the acquisition of data using a frequency-modulated continuous-wave (FMCW) radar. Then, it proceeds with signal processing to generate three different radar domain representations, which are then sequentially applied to the four different CNN models. The following subsections explain each component of the system model in detail.

### 3.1. Data Acquisition

This study used a dataset from the James Watt School of Engineering at the University of Glasgow, UK, which includes a wide range of everyday human movement activities [44], as described in Table 2. The selected dataset is notable for its extensive use in recent academic work [45], which ensures that our research is consistent with current trends in the field. The dataset was collected using a single-input and single-output (SISO), frequency-modulated continuous-wave (FMCW) radar operating at a 5.8 GHz carrier frequency and a chirp bandwidth of 400 MHz, involving 81 volunteers of varying ages. The radar dataset comprises the following parameters: Each chirp lasts 1 ms and contains 128 ADC samples at a sampling rate of 128 kHz. The pulse repetition frequency (PRF) is 1 KHz. The range resolution is 37.5 cm, while the Doppler resolution is 1.25 Hz, corresponding to a velocity resolution of 3.2 cm/s.

#### FMCW Radar Principle

In FMCW radar, a chirp signal with slope is transmitted from the broadcast antenna using a carrier frequency, $f_{c}$. An FMCW radar can capture the detailed time, range, and velocity (Doppler) information of a subject, making it a valuable tool in HAR systems. The fundamental representation of a radar signal reflecting towards a target is given by [46]:

(1) $$s_{t}\left(t\right)=\sin\left(2\pi f_{c}t+\pi \alpha t^{2}\right)$$

Here, α, which defines the slope, can be represented as follows:

(2) $$\alpha =\frac{\Omega }{T_{c}},$$

where $T_{c}$ is the chirp duration. The range resolution $R_{\mathrm{res}}$ is impacted by the chirp bandwidth, Ω, and the speed of light, *c*, as follows:

(3) $$R_{\mathrm{res}}=\frac{c}{2\Omega }$$

The received signal $s_{r}\left(t\right)$, corresponding to an attenuated and delayed $T_{d}$ copy of the transmitted signal $s_{t}\left(t\right)$, reflects from a target positioned at distance *d* and is given by the following:

(4) $$s_{r}\left(t\right)=\xi s_{t}\left(t-T_{d}\right)$$

Here ξ, represents the attenuation coefficient. The parameter $T_{d}$ is determined by the round-trip delay of the target from the radar and is given by the following:

(5) $$T_{d}=\frac{2R}{c},$$

where *R* is the range to the target. The replica of $s_{r}\left(t\right)$ captured via the received antenna is mixed with the $s_{t}\left(t\right)$ to produce an intermediate frequency (IF) signal by applying a low-pass filter within the radar equipment, which removes the frequency component $f_{c}$, and this IF signal is expressed as follows:

(6) $$y\left(t\right)=-\frac{\xi }{2}\cos\left(2\pi \alpha T_{d}t+2\pi f_{c}T_{d}-\pi \alpha T_{d}^{2}\right)$$

The spectrum of the baseband signal exhibits peaks at specific frequencies that correspond to the range between the nearby objects and radar sensor because its frequency is proportional to $T_{d}$ and, consequently, *R*. This is because the received signal is essentially the sum of multiple signals of the specified type shown in Equation (6), when there are multiple targets. The analog-to-digital converter (ADC) changes the in-phase signal from analog to digital, resulting in the discrete-time signal y[i], and it is determined as

(7) $$y\left[i\right]=y\left(t=iT_{s}\right)$$

The maximum range, $R_{\max}$, covered by the radar is affected by the choice of sampling period, $T_{s}$, of the ADC [46]:

(8) $$R_{\max}=\frac{c}{2\alpha T_{s}}$$

The result (Equation (7)) is then demodulated to baseband to produce the in-phase (I) and quadrature (Q) components. The beat signal’s frequency is proportional to the target range, while its phase provides information about the target’s velocity (Doppler effect). Analyzing the I and Q signals allows the determination of both the range and velocity of the target. The IF signal is organized in such a way that its rows and columns correspond to slow and fast time variables, respectively, where “fast time” refers to the time of a single sweep, whereas “slow time” spans across multiple sweeps [24].

### 3.2. Data Preprocessing

The IF signal that contains the I and Q signals undergoes several steps to improve its quality and accurately extract human activity information, as shown in Figure 2. First, these signals were converted into a complex digital format and reshaped into a 2D matrix to align the data for subsequent processing before the fast Fourier transform was applied (FFT).

#### 3.2.1. Range-FFT-Based Time-Range (TR) Maps

The process begins by applying the Hamming window to minimize spectral leakage, followed by conducting an FFT on the fast-time axis (also referred to as the number of ADC samples per chirp) to extract range information over time, known as Range-FFT or range profile. The range profile $X_{n}\left[k\right]$, for chirps $n=1,2,\ldots ,N_{tot}$ ($N_{tot}$ is the total number of chirps), is defined as the discrete Fourier transform (DFT) of y[i]. For the presence of a target, we examine the peaks in the magnitude of $X_{n}\left[k\right]$, which is acquired via DFT as [46]:

(9) $$X_{n}\left[k\right]=\sum_{i=0}^{N}w\left[i\right]y_{n}\left[i\right]e^{-j2\pi \frac{ki}{N}}$$

where N=128 is the number of ADC samples of the received IF signal $y_{n}$ for chirp *n*, *k* shows the frequency bin, and *w* shows the window function, which is hamming in our case.

**Figure 3:**
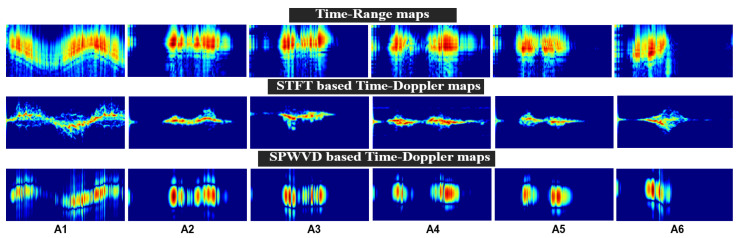
Two-dimensional images of six activities resulting from TR, STFT, and SPWVD techniques.

The time-range (TR) domain reflects the time-varying range information between the radar and the target. A filter called a moving target indicator (MTI) detects only moving targets and effectively removes any clutter or stationary objects from the radar signal. The MTI filter was designed as a fourth-order Butterworth high-pass filter with a cut-off frequency of 0.0075 Hz. After filtering with MTI, the range profiles or TR maps are obtained, as shown in Figure 3. By visually inspecting these maps, we identified the range bins of interest, $k^{*}$, where the targets are predominantly detected. Finally, the STFT and SPWVD techniques were applied to these selected range bins, $k^{*}$, to generate time-Doppler (TD) maps, as detailed in the subsequent subsections and illustrated in Figure 3.

#### 3.2.2. STFT-Based TD Maps

To obtain a TD representation of the activity, we employed the STFT technique. In particular, the STFT employs a Hann window of size 200, with the FFT incorporating 800 sampling points, a zero padding factor of 4, and a 95% overlap between consecutive frames (i.e., 190 samples). This approach balances frequency and time information, resulting in a 2D image, as shown in Figure 3. The mathematical representation of the STFT applied to the selected range bins of interest $k^{*}$ from the range profile $X_{n}\left[k^{*}\right]$ is given by [38]:

(10) $$\Theta \left[m,f\right]=\sum_{n=-\infty }^{\infty }X_{n}\left[k^{*}\right]g_{s}\left[n-mR\right]e^{-j2\pi fn}$$

where $k^{*}$ denotes the range bins of interest (5 to 25), and $g_{s}$ represents the window function, which is a Hann window with a segment length of s=200 samples and a hope size of R=10 samples. Figure 3 actualy shows the STFT-based TD spectrograms, which were generated by taking the magnitude of Θ[m,f].

The time-frequency resolution of the STFT domain is contingent upon the selection of the window function. An extended window increases the frequency resolution, but a reduced window maximizes the time resolution. As a result, the STFT experiences a trade-off, rendering it unable to achieve high resolution in both the time and frequency domains concurrently.

#### 3.2.3. SPWVD-Based TD Maps

Since STFT is limited by non-independent time and frequency windows, SPWVD provides a more sophisticated approach to TD analysis by utilizing independent windows. SPWVD is a subset of the Wigner–Ville distribution (WVD), which offers a high time-frequency resolution but suffers from cross-term artifacts. SPWVD addresses this limitation by applying smoothing operations in both the time and frequency domains, effectively eliminating the cross terms in both dimensions. This dual smoothing operation makes SPWVD advantageous for WVD by providing a cleaner time-frequency representation. SPWVD is also applied to the extracted range bins of interest, $X_{n}\left[k^{*}\right]$, and is mathematically expressed as follows [38]:

(11) $$\Theta \left[m,f\right]=\sum_{n=-\infty }^{+\infty }\sum_{\tau =-\infty }^{+\infty }X\left(k^{*}+\frac{\tau }{2},n\right)X\left(k^{*}-\frac{\tau }{2},n\right)\times h\left(\tau \right)w\left(n-m\right)e^{-j2\pi f\tau }$$

Here, $\pm \frac{\tau }{2}$, represents the symmetrical time shifts used for cross-term smoothing in the SPWVD. For the frequency smoothing window h(τ), we employed a Hann window with a length of 15, whereas for the time smoothing window w(n−m), we used a Kaiser window with a length of 25, and *m* is the time index. To further reduce artifacts and avoid intensity spikes, the SPWVD outputs were normalized by the number of range bins. This comprehensive approach provides detailed TD-based SPWVD representations, as shown in Figure 3.

One of the objectives of this study is to evaluate the applicability of the SPWVD as a spectrogram technique and to examine its characteristics to determine the potential benefits for efficient HAR systems. Therefore, integrating these three domains, utilizing their respective strengths, and addressing their challenges are essential to improve the performance of the HAR system. This comprehensive approach ensures that the data fed into the CNN architecture are well suited for learning and recognizing activity-specific features, thus achieving a balance between processing efficiency and performance accuracy.

### 3.3. Training Pipeline and Optimization

Following the discussion in the previous section, Section 3.2, we preprocessed the acquired data and utilized pre-trained models, as shown in Figure 2. This process involves three steps: preparing the data for input to the CNN, selecting and optimizing the model, and training the data on the selected model.

#### 3.3.1. Data Preparation

The first step of the training phase is preprocessing to standardize all images in the dataset. Each image was resized to a uniform size of 224 × 224 pixels with three channels (RGB) to satisfy the input size requirement of the selected CNN model. This preprocessing step also includes image normalization and the subtraction of the mean RGB value based on the training set, as well as other necessary transformations. To ensure a strong learning environment, the processed images were labeled and randomly shuffled. This process was designed to ensure a diverse and representative distribution of data within the dataset. In terms of data allocation, we adopted an 80–20 split; 80% of the data were used for training, and the remaining 20% were used for testing purposes. An important aspect of our dataset management method is the stratified partitioning approach, which ensures that the class distribution remains consistent in the training and test sets.

#### 3.3.2. CNN Pre-Trained Models

The basis of our approach is to apply the transfer learning (TL) method. TL is a powerful technique in deep learning (DL), in which a model developed for one task is reused as a starting point for a model for a second task. It is particularly useful in scenarios with limited labeled data, such as radar datasets for HAR. Hence, it is not necessary to train a CNN from scratch. In addition, it plays a key role in alleviating overfitting and enhancing the generalization process. This approach involves leveraging pre-trained weights from widespread datasets, such as ImageNet [47], which is particularly useful in addressing the class imbalance problem in the dataset used. In this study, we investigated the effectiveness of four prominent TL-based CNN architectures: VGG-16, VGG-19, ResNet-50, and MobileNetV2, as shown in Figure 2.

**Visual Geometry Group (VGG):** VGG-16 and VGG-19 are CNN architectures developed by the Visual Geometry Group (VGG) at the University of Oxford, UK [48]. The VGG-16 architecture contains 16 layers and is known for its simplicity and performance in image recognition tasks. The model achieved a top-five test accuracy of nearly 92.7% on ImageNet. It replaces filters with large kernel sizes with several 3 × 3 kernel-size filters, providing a significant improvement over the AlexNet model. The same group extended the VGG-16 to VGG-19 [49]. The numbers ‘16’ and ‘19’ denote the weight layers. Although similar in structure to VGG-16, the added layers provide deeper features that may improve the recognition performance for complex scenes in radar-based HAR datasets.

**ResNet-50:** The ResNet architecture had multiple configurations, each with a different number of layers. In our study, we chose to implement ResNet-50, a variant of ResNet equipped with 50 neural network layers [50]. The ResNet-50 model stands out for its ability to handle CNN tasks without performance degradation, which is a common problem when scaling CNN structures. With its 50-layer framework, ResNet-50 excels at recognizing complex patterns and performs well across a range of recognition tasks. This deep architectural capability is particularly beneficial for detecting subtle human activities in radar data.

**MobileNetV2:** MobileNetV2 is a successor to the original MobileNet [51], and it is a CNN variant designed specifically for mobile and embedded vision applications. This architecture ensures that the network is not only lightweight but also has a lower inference latency, which is crucial for critical applications such as fall detection. MobileNetV2 uses two unique block structures: a residual block with stride 1 to maintain dimensionality, and another block with stride 2 to reduce space [34]. Despite its light weight, MobileNetV2 is expected to maintain a competitive performance compared to models such as VGGs or ResNet.

#### 3.3.3. Model Optimization

When the CNN models were optimized, a grid search was used to determine the best hyperparameters that achieved a high classification accuracy for all classes. The grid search explored parameters such as the learning rate, batch size, dropout rate, and number of neurons in the dense layers. During the grid search, data samples from each radar domain (TR, STFT, and SPWVD) were utilized independently. For each domain, a train–test split approach was applied, with 15% of the training dataset aside as a validation set to determine the optimal hyperparameters. This protocol was repeated individually for the TR, STFT, and SPWVD domains to ensure that the grid search process was tailored to the unique characteristics of each domain. Consistency was maintained across all the radar domains and model architectures (VGG-16, VGG-19, ResNet-50, and MobileNetV2). To mitigate overfitting and minimize loss, batch normalization and dropout layers were strategically placed before the flattening layer. Table 3, provides a comprehensive summary of the best parameters for each model that were found during grid searching.

The models contained a dense layer with 512 neurons for VGG-16 and 1024 neurons for VGG-19 and ResNet-50. The layers were initialized using *ReLU* activation and the *he_normal* kernel initialization function. To enhance the generalization, an additional dropout layer was added after the last dense layer. The number of neurons was adjusted according to the unique characteristics of each type of 2D domain: 2048 neurons were used for the noisy TR domain, 512 neurons were used for the high-definition SPWVD, and 1024 neurons were used for the STFT, which is known for its medium clarity and complexity. These adjustments aimed to reduce overfitting while optimizing model performance, adapting to the specific characteristics of each spectrogram. All CNN models adopted *categorical cross-entropy* as the loss function, prioritizing accuracy optimization.

For MobileNetV2, a similar grid search approach determined the ideal number of neurons in dense layers and was tailored for each radar preprocessing technique with consistent hyperparameters in the VGG and ResNet-50 models. Modifications to the optimizer and the learning rate are presented in Table 3. From prior research [52], it was observed that the Adam optimizer was more effective than SGD for radar-based representations using mobileNetV2. This informed our choice of the optimizer, while the learning rate was determined through our grid search results. When we trained MobileNetV2 using the same parameters as the other network models, the model achieved good accuracy, but it exhibited overfitting. The adjusted learning rate and optimizer help mitigate this issue, ensuring better generalization, as demonstrated in the experimental results.

#### 3.3.4. Model Training

To optimize training robustness, we used a stratified 15 k-fold cross-validation strategy on the training data, which comprised 80% of the entire dataset. For each fold, the training data were divided into 15 subsets with an even class distribution. Out of the 15 subsets, 14 subsets (90% of the fold) were used for training, whereas one subset (10% of the fold) was used for validation. The performance of each hyperparameter configuration was evaluated based on the validation accuracy across all 15 folds. This approach ensures consistency between the grid search and the training–validation split described in Section 3.3.3. For each fold, the training duration and validation accuracy were monitored. The training process consisted of fitting the model to the training data using the validation split and saving the model weights with the highest validation accuracy across all folds.

#### 3.3.5. Performance Evaluation

Our methodology for evaluating the performance of the proposed radar domain in conjunction with CNN models includes four key metrics: accuracy, precision, recall, and F1 score. These metrics are calculated by comparing the predicted and actual results, resulting in four possible returns: true positive (TP), true negative (TN), false positive (FP), and false negative (FN).

Accuracy represents the overall effectiveness of the model. It is defined as the ratio of correct predictions, including both TP and TN, to all predictions made. Mathematically, it is expressed as follows:

(12) $$Accuracy=\frac{TP+TN}{TP+FP+TN+FN}\times 100\%$$

Precision measures the reliability of the model in classifying an instance as positive. It focuses on the proportion of TP relative to all positive predictions, which include both TP and FP. Precision is particularly important in minimizing Type-1 errors (FP). Mathematically, it can expressed as follows:

(13) $$Precision=\frac{TP}{TP+FP}$$

Recall quantifies the model’s ability to correctly identify TP instances. It is the proportion of actual positives that are correctly predicted as positive. The mathematical representation of recall is as follows:

(14) $$Recall=\frac{TP}{TP+FN}$$

The F1 score is a harmonic mean of recall and precision, providing a balanced measure of both metrics. It is particularly useful for assessing the model’s ability to handle imbalanced classes by balancing Type-I and Type-II errors. A decrease in either precision or recall affects the F1 score, highlighting its value as a preferred metric for evaluation in many classification problems. Formally, the F1 score is given by the following:

(15) $$F1\text{-}\mathrm{Score}=\frac{2\cdot (Recall\cdot Precision)}{Precision+Recall}$$

#### 3.3.6. Computational Efficiency

When evaluating 12-MDPs, we consider not only accuracy or F1 score but also computational efficiency, which is critical in real-time applications. In this context, the key factors for computational efficiency are training time and inference time, which are evaluated throughout the model training and prediction stages:

- **Training time:** This is the time required to train the model using a particular radar-based domain. Training time is an important parameter because extended training can be difficult in cases where models need frequent updates or computing resources are limited. Achieving fast training times improves the utility of the model in a range of applications.
- **Inference time:** To measure the inference time ($T_{\mathrm{inference}}$) of the model, we adopted the simple method from [53], focusing on the time required to perform a single inference cycle on the test set. Specifically, the inference time is calculated as follows:
  (16) $$T_{\mathrm{inference}}=T_{\mathrm{end}}-T_{\mathrm{start}}$$
  where $T_{\mathrm{start}}$ represents the timestamp when the inference request is issued, and $T_{\mathrm{end}}$ represents the timestamp when the inference result is obtained.

### 3.4. Proposed Radar-Based HAR Algorithm

Algorithm 1 outlines the main components of the proposed radar-based HAR system to provide a brief overview of our technique. This includes data acquisition, preprocessing, model selection and training, and activity classification.    
**Algorithm 1:** Proposed radar-based HAR system**Require:** Raw radar data y[i]
**Ensure:** Classified human activities  1:**procedure** Data Acquisition and Preprocessing  2:    Collect raw radar data {y[i]} and convert to complex signal $s\left[i\right]=y\left[i\right]+j\cdot \hat{y}\left[i\right]$  3:    Reshape s[i]→S, a 2D matrix of size (N,M)  4:    Apply Hamming window w[n]: $S_{w}\left[i\right]=S\left[i\right]\cdot w\left[n\right]$  5:    Apply IIR Notch MTI filter: $S_{\mathrm{filtered}}\left[i\right]=\mathrm{IIR}\left(S_{w}\left[i\right]\right)$  6:    Perform Range-FFT: $X_{n}\left[k\right]=\mathrm{FFT}\left(S_{\mathrm{filtered}}\left[i\right]\right)$  7:    Extract desired range bins: $R=X_{n}\left[k\right],\mathrm{for}\;k\in \mathrm{Range}\;\mathrm{bins}$  8:    **Choose Preprocessing Method:**  9:       **Option 1:** Generate Time-Range (TR) maps: TR(t,r)10:       **Option 2:** Apply STFT: TD(t,f)=STFT(R)11:       **Option 3:** Apply SPWVD: ${\mathrm{TD}}_{\mathrm{high}\text{-}\mathrm{res}}\left(t,f\right)=\mathrm{SPWVD}\left(R\right)$12:    Resize images to 224×224 and normalize: $I=\mathrm{Norm}(I_{224\times 224})$13:    Split data: $I_{\mathrm{train}},I_{\mathrm{test}}\leftarrow \mathrm{Split}\left(I\right)$14:**end procedure**15:**procedure** Model Selection and Training16:    **Choose CNN base model:**17:       BaseModel∈{VGG-16,VGG-19,ResNet-50,MobileNetV2}18:    Load pre-trained weights: BaseModelWeights←Pre-trained19:    Inputs: x→BaseModelInput20:    **Add Custom Layers:**21:    z←Dense(N,activation=‘softmax’)        ▹*N*: number of activity classes          22:    Define Model: M=Model(input=x,output=z)23:    Compile Model: M←Adam(α=0.0004),Loss=CategoricalCrossentropy24:    Train Model: $M_{\mathrm{trained}}\leftarrow \mathrm{Train}\left(M,I_{\mathrm{train}}\right)$25:    Estimate Training Time: $T_{\mathrm{training}}=\mathrm{End}\;\mathrm{Time}-\mathrm{Start}\;\mathrm{Time}$26:    Save Model Weights: $W^{*}\leftarrow W_{t},\;\mathrm{where}\;A_{\mathrm{val},t}=\max\left\{A_{\mathrm{val},1},\cdots ,A_{\mathrm{val},T}\right\}$27:**end procedure**28:**procedure **Activity Classification29:    Input new radar data $y_{\mathrm{new}}$30:    Preprocess: $I_{\mathrm{new}}=\mathrm{Preprocess}\left(y_{\mathrm{new}}\right)$31:    Predict Activity: $\hat{a}=M_{\mathrm{trained}}\left(I_{\mathrm{new}}\right)$32:    Output Classified Activity: $a=\arg\max(\hat{a})$33:    Estimate Inference Time: $T_{\mathrm{inference}}=\mathrm{End}\;\mathrm{Time}-\mathrm{Start}\;\mathrm{Time}$34:**end procedure**

### 3.5. Runtime Environment

In our study, we performed data preprocessing on raw radar data and CNN training using Python 3.9.0 toolkits on a GPU-accelerated PC. The system was equipped with an 11th-generation Intel^®^ Core™ i7-11700 processor with 8 cores and 16 threads enabled by hyper-threading technology and a base frequency of 2.50 GHz. It also came with 16 GB of RAM and an NVIDIA GeForce RTX 3060 Ti graphics card with 8 GB of memory. To generate radar spectra, we used Python libraries such as Scipy for signal processing, time-frequency for spectrum analysis, and fftpack for FFT execution. For CNN model training, we used the Keras 2.8.0 and TensorFlow 2.10.0 frameworks, taking advantage of the multi-core and multi-threaded CPU capabilities of the workstation and the parallel processing power of the GPU.

## 4. Results and Discussion

In this section, we present a detailed analysis of the results for each CNN model discussed in Section 3.3.2, using the radar maps described in Section 3.2 as inputs. The results focus on both performance evaluation and computational efficiency, particularly examining how multiple radar maps contribute to the models’ ability to extract relevant features essential for HAR systems in real-world deployment. To ensure stable and reliable measurements, the experimental results include recognition accuracy, which remains constant across runs, owing to the use of saved models for testing on the 20% test data set aside before training, as discussed in Section 3.3.4. The inference times were averaged over five runs to account for computational variability.

### 4.1. Performance Comparison of Proposed HAR Models

**Figure 4:**
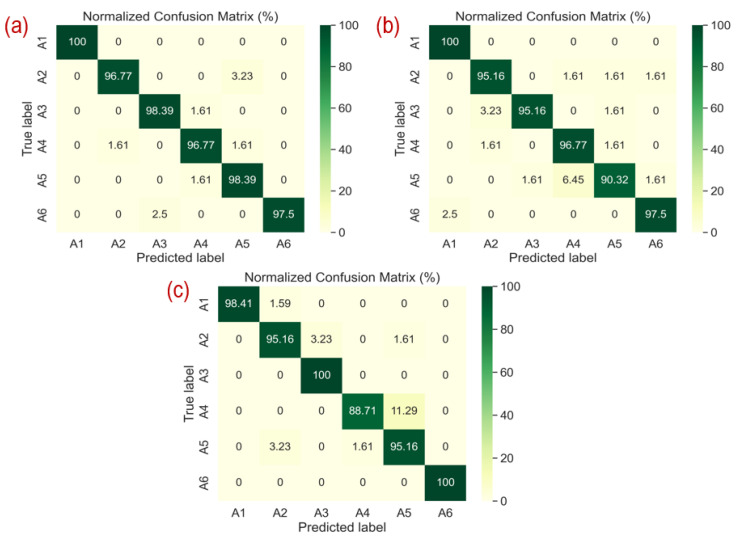
Confusion matrices of best-performing pairs. (**a**) shows pair M1, (**b**) shows pair M7, and (**c**) shows pair M10.

In this section, we will compare the performance metrics of the 12 MDPs (named M1, M2, M3, etc.), as described in Section 3.3.5, and the results are listed in Table 4. Based on these metrics, the M1, M7, and M10 pairs were selected from the 12-MDPs as the best-performing pairs in terms of accuracy. The M1, M7, and M10 pairs achieved the highest recognition accuracy in their respective radar domains (TR, STFT, and SPWVD) when used as input, confirming their importance for radar-based HAR systems. To evaluate the classification performance of each class, the confusion matrices were analyzed. Figure 4a–c show the confusion matrices for pairs M1, M7, and M10 that perform best. In particular, pair M7 identified A6, representing fall activity, with 100% accuracy, whereas pairs M1 and M10 achieved 97.50% accuracy. The three pairs, except pair M10, detected the A1 class, which represents walking activity, with 100% accuracy and 98.41% for pair M10.

The performance differences observed for certain classes, such as A4 (bend to pick an object) and A5 (drinking water), were primarily due to the similar motion patterns exhibited by these activities in the radar domain representations, as shown in Figure 4. Both activities involve localized arm movements, making them challenging to differentiate, particularly in radar domains, such as SPWVD and STFT, which may amplify overlapping features or lack sufficient resolution to distinguish fine-grained differences. This is evident in MDPs like M10 for A4 and M7 for A5, where misclassification rates are higher. These observations suggest that the nature of the activities and radar preprocessing techniques play an important role in the classification performance.

### 4.2. Generalization Performance of HAR Models

**Figure 5:**
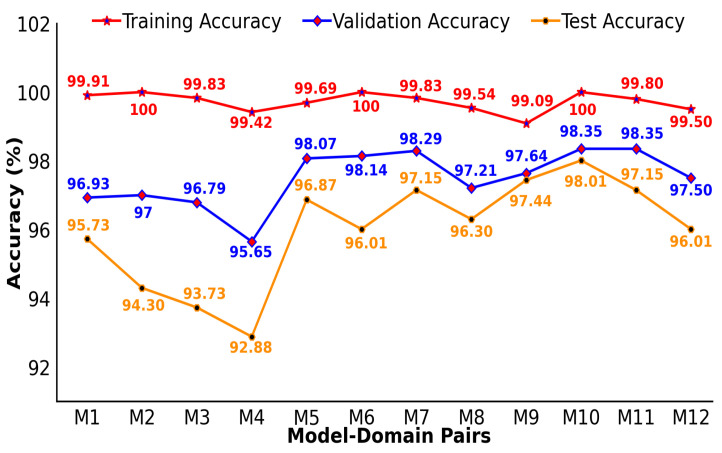
Generalization capability of the proposed HAR system.

Evaluating the generalization ability of HAR systems is essential, particularly for limited datasets on radar-based human activity. Due to a lack of data, CNN classifiers tend to overfit. Therefore, it is important to evaluate the model’s performance on new, unseen radar data, which constitute the remaining 20% test set. The evaluation results for our HAR models are shown in Figure 5, showing consistent results, with the test accuracy showing the smallest variance between the 12 MDPs, as shown in Table 4.

In Figure 5, the findings indicate that the models have strong generalization capabilities, with test accuracies ranging from 92.88% to 98.01%, confirming their effectiveness on the new data. Pair M10 achieved the highest test accuracy of 98.01%. On the other hand, although pairs M2 and M4 achieved perfect or near-perfect average training accuracies of 100% and 99.42%, respectively, they had lower test accuracies of 94.30% and 92.88%, indicating that there is room for improvement. This difference highlights the importance of the thorough testing of unseen data to accurately determine how well a model adapts to new input. The results emphasize the importance of using cross-validation and remaining datasets for testing to evaluate model generalization in real-world scenarios. Furthermore, choosing the best MDP requires a balanced evaluation of its performance, based on its generalization ability and computational efficiency.

### 4.3. Computational Efficient and Lightweight HAR Model

Computational efficiency is critical for real-time radar-based HAR system, particularly for resource-constrained edge devices. Therefore, it is crucial to develop a lightweight model that can quickly and accurately recognize human activities while minimizing the inference latency. This section examines computational efficiency using time metrics, such as training time and inference time as defined in Section 3.3.6, to evaluate the suitability of various models for real-world deployment, as detailed in Table 5. The fastest predicted pairs, M4, M8, and M12, were chosen based on inference time, an important metric for resource-constrained edge devices that require fast activity prediction.

The inference time is also defined as the time between initiating a prediction request and receiving the prediction output from the test model. This metric is very important for evaluating the performance and efficiency of a model, particularly in applications that require real-time processing on edge devices or standalone systems. Inference time directly affects the user experience and applicability of the model in time-sensitive scenarios, such as fall detection.

### 4.4. Computational Cost Across Radar Domains

**Figure 6:**
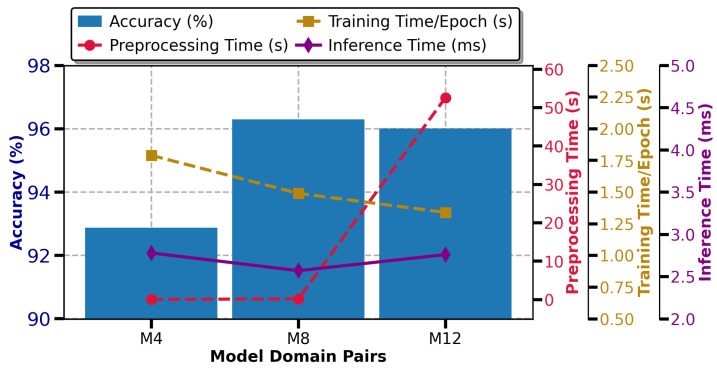
Performance and computational analysis comparison across radar domains as input to MobileNetV2.

For the TR domain, the preprocessing time for processing the input raw radar data and visualizing an image representing the range over time, as shown in Figure 3, is only 0.035 s, which is very low compared to other techniques illustrated in Figure 6. However, when inputted to a CNN model, it results in lower accuracy and higher computational cost. Among all models, MobileNetV2 (M4) exhibits the best training and inference efficiency, with a training time of 1.79 s/epoch, an inference time of 2.78 ms/sample, and a recognition accuracy of 92.88%, as shown in Figure 6. In contrast, the other three models (VGG-16, VGG-19, and ResNet-50) are known for their higher accuracy but with increased time measurements when using the TR domain as the input.

The STFT-based TD map shows the change in frequency over time (Doppler shift), as shown in Figure 3, and it takes only 0.22 s to preprocess and generate a spectrogram using the STFT method. When this spectrogram is used as an input feature to the network, it provides a good balance between performance and efficiency. For example, MobileNetV2 (M8) achieved a training time of 1.49 s/epoch and an inference time of 2.57 ms/sample, with a test accuracy of 96.30%. In contrast, VGG-16 and ResNet-50 achieved higher recognition accuracies of 96.87% and 97.15%, respectively, but they had longer training and inference times, indicating higher resource usage, as illustrated in Table 4 and Table 5. On the other hand, VGG-19 achieved a longer prediction time of 6.90 ms/sample, making it less suitable for real-time systems.

Despite the lengthy preprocessing time of 52.58 s to generate a spectrogram using the SPWVD method, MobileNetV2 (M12) achieved a lower training time of 1.34 s/epoch and an inference time of 2.76 ms/sample with a recognition accuracy of 96.01%, as shown in Figure 6, when SPWVD was used as input compared to the TR and STFT domains. This shows the advantage of a higher resolution by combining both time and frequency windows simultaneously, as detailed in Section 3.2.3. Despite the advantage, SPWVD is still not suitable for real-time systems due to the higher preprocessing time that requires rapid preprocessing and a fast prediction response, from data acquisition to model prediction specifically for critical activity like fall detection in care homes for elders.

### 4.5. Comprehensive Evaluation of Model–Domain Pairs

This study highlights two key possibilities for selecting the optimal radar domain and model combination for HAR systems. Each radar domain demonstrates unique strengths and limitations, making it suitable for specific applications, depending on the recognition accuracy and computational efficiency requirements.

The TR maps are computationally inexpensive to preprocess (0.035 s), and they represent the range of the target with respect to time after applying a single FFT with a Hamming window and MTI filter. These maps include all range bins derived from FFT, often retaining noisy and extraneous data. In contrast, STFT and SPWVD were applied to the selected range bins, focusing on areas where the target was detected, as identified from the TR maps. This selective approach enhances the feature extraction by isolating target-specific data. However, TR maps also lack Doppler information, which limits their ability to capture richer activity features. Among the models tested with TR maps, VGG-16 achieved the highest accuracy of 95.73% by effectively handling noise but at the cost of higher training and inference times, as shown in Table 4 and Table 5. In contrast, MobileNetV2 provided faster training and inference times, achieving an accuracy of 92.88%, making it more suitable for less-demanding real-time applications. These findings underscore the importance of frequency-based domains in radar-based HAR systems for achieving higher recognition accuracy and lower computational costs.

Doppler-based domains, such as STFT and SPWVD, achieved better time measurements, making them suitable for resource-constrained devices. SPWVD derived TD maps paired with the VGG-19 model achieved the highest accuracy of 98.01% with 100% classification for fall activity, making it ideal for applications that prioritize accuracy over computational cost, such as detailed behavioral analysis or offline systems. However, the significant preprocessing time (52.58 s) and increased computational cost render this combination less viable for real-time systems, where rapid data processing and prediction are significant.

In contrast, STFT-derived TD radar maps strike a balance between computational efficiency and accuracy. When paired with MobileNetV2, this combination achieved a remarkable accuracy of 96.30% with the shortest inference time of 2.57 ms/sample. This makes STFT-MobileNetV2 particularly suited for real-time applications such as fall detection in healthcare or activity monitoring in edge computing environments. The moderate preprocessing time (0.22 s) further enhances its practicality for latency-sensitive tasks.

However, the study had limitations. A key challenge is the need for more studies on energy consumption, which are required to implement these models in resource-constrained edge devices. Future research should incorporate energy consumption measures to assess the suitability of each model and radar domain combination in low-power environments. In addition, further studies might look at how model reduction and optimization approaches, such as quantization and pruning, can improve the deployment potential of radar-based HAR systems on edge devices.

### 4.6. Comparison of Pair M8 with State-of-the-Art Models

A detailed comparative analysis is presented in Table 6. All models used STFT-based spectrogram inputs with a resolution of 224 × 224 pixels. The CNN [54] model trained from scratch achieved 95.44% accuracy, but the inference time per sample was 5.14 ms, which is almost twice that of our proposed TL-based model MobileNetv2 model. The CNN + LSTM [24] model achieved the shortest training time per epoch of 1.12 s, but the accuracy was only 84.90%, and the inference time was as high as 6.04 ms/sample, almost three times that of our proposed model. The Bi-LSTM [43] model achieved a competitive accuracy of 95.16% with an inference time of 2.77 ms/sample, lagging behind the MobileNetV2 model in both training and inference time.

The proposed MobileNetv2 model (Pair M8), leveraging TL, achieved the highest accuracy of 96.30% and the best inference time of 2.57 ms/sample, making it suitable for real-time applications. This shows that MobileNetV2 not only surpasses the accuracy of other state-of-the-art models trained from scratch but also significantly reduces inference time, providing an efficient option for real-time processing. This comparison highlights the adaptability and potential of our proposed work for a wide range of future applications, setting a benchmark for HAR systems in terms of both performance and efficiency.

## 5. Conclusions

In this study, we applied three preprocessing techniques, particularly Range-FFT for TR, STFT, and SPWVD, as inputs to CNN models for HAR, and we evaluated their computational efficiency for edge deployment, resulting in twelve different combinations of model-preprocessing pairs. These combinations include VGG-16, VGG-19, ResNet-50, and MobileNetV2 architectures. Among them, the combination of MobileNetV2 with STFT (model M8) showed balanced performance, setting a new benchmark for the state-of-the-art radar-based HAR system. This result emphasizes the importance of a thorough evaluation of the entire process chain. The effectiveness of model M8 highlights its ability to support more advanced edge device models, which are typically associated with TinyML. Our work not only contributes to current methodologies but also lays the foundation for integrating more complex models into low-power, real-time edge systems.

Furthermore, in anticipation of advancements, our future research will focus on integrating neuromorphic federated learning and congestion-aware spiking neural networks to design energy-efficient systems, which is an important aspect not discussed in this study. This strategy aims to improve the real-time performance of radar-based HAR systems and address the trade-off between accuracy and energy efficiency.

## Figures and Tables

**Table 1:** Comparison of key characteristics and attributes among existing state-of-the-art radar-based HAR studies.

Ref.	Radar Domains			Data Preprocessing	CNN/LSTM	TL-Based
	TR	STFT	SPWVD	Time		Methods
[16]	✗	✓	✗	✗	✓	✗
[23]	✓	✓	✗	✗	✓	✗
[24]	✓	✓	✗	✗	✓	✗
[25]	✗	✓	✓	✗	✗	✓
[26]	✗	✓	✓	✗	✓	✗
[38]	✗	✓	✓	✗	✓	✗
[41]	✗	✓	✗	✗	✓	✗
[42]	✗	✓	✗	✗	✗	✓
[43]	✗	✓	✗	✗	✓	✗
**Our**	✓	✓	✓	✓	✓	✓

**Table 2:** Details of human activity classes.

Short Name	Activity Description	Samples	Duration
A1	Walking back and forth	312	10 s
A2	Sitting on a chair	311	5 s
A3	Standing	311	5 s
A4	Bend to pick an object	309	5 s
A5	Drinking water	311	5 s
A6	Fall	197	5 s

**Table 3:** Pre-trained CNN hyperparameters.

Parameters	VGG-16	VGG-19	ResNet-50	MobileNetV2
Batch size	32	32	32	32
Dropout	0.5	0.2	0.2	0.2
Learning rate	2 $\times \;10^{-3}$	2 $\times \;10^{-3}$	2 $\times \;10^{-3}$	4 $\times \;10^{-4}$
Optimizer	SGD	SGD	SGD	Adam
Decay	-	1 $\times \;10^{-5}$	1 $\times \;10^{-6}$	-
Momentum	0.9	0.9	0.9	-
Epochs/fold	25	25	25	25

**Table 4:** Comparison of evaluation metrics for proposed HAR models across radar domains.

MDPs	Radar Domains	Models	Accuracy (%)	Precision	Recall	F1 Score
**M1**	**TR**	**VGG-16**	**95.73**	**0.9576**	**0.9573**	**0.9572**
M2	TR	VGG-19	94.30	0.9436	0.9430	0.9429
M3	TR	ResNet-50	93.73	0.9373	0.9373	0.9368
M4	TR	MobileNetV2	92.88	0.9307	0.9288	0.9284
M5	STFT	VGG-16	96.87	0.9697	0.9687	0.9687
M6	STFT	VGG-19	96.01	0.9639	0.9624	0.9624
**M7**	**STFT**	**ResNet-50**	**97.15**	**0.9721**	**0.9731**	**0.9721**
M8	STFT	MobileNetV2	96.30	0.9635	0.9651	0.9642
M9	SPWVD	VGG-16	97.44	0.9764	0.9744	0.9745
**M10**	**SPWVD**	**VGG-19**	**98.01**	**0.9803**	**0.9801**	**0.9801**
M11	SPWVD	ResNet-50	97.15	0.9720	0.9715	0.9715
M12	SPWVD	MobileNetV2	96.01	0.9629	0.9580	0.9600

**Table 5:** Comparison of training and inference times for computational efficiency across 12 MDPs.

Model–Domain	Training	Inference
Pairs	Time/Epoch (s)	Time/Sample (ms)
M1	3.40	7.16
M2	3.77	8.11
M3	2.77	3.80
**M4**	**1.79**	**2.78**
M5	3.38	7.10
M6	4.38	6.90
M7	2.74	3.54
**M8**	**1.49**	**2.57**
M9	3.50	7.02
M10	3.76	6.88
M11	2.73	3.99
**M12**	**1.34**	**2.76**

**Table 6:** Time metrics and accuracy comparison of proposed lightweight model against alternative approaches.

Ref.	Models	Training	Inference	Accuracy
		Time/Epoch (s)	Time/Sample (ms)	(%)
[24]	CNN+LSTM	1.12	6.04	84.90
[43]	Bi-LSTM	1.70	2.77	95.16
[54]	CNN	1.88	5.14	95.44
Our	**Pair M8**	**1.49**	**2.57**	**96.30**

## Data Availability

This study was conducted using a publicly available dataset. Here is a link to the dataset: https://researchdata.gla.ac.uk/848/ (accessed on 5 December 2024).
